# Development of an Innovative Pupillometer Able to Selectively Stimulate the Eye’s Fundus Photoreceptor Cells

**DOI:** 10.3390/diagnostics14171940

**Published:** 2024-09-02

**Authors:** Giovanni Gibertoni, Anton Hromov, Filippo Piffaretti, Martial H. Geiser

**Affiliations:** 1 Department of Engineering “Enzo Ferrari”, University of Modena and Reggio Emilia, 41125 Modena, Italy; 2 Oculox Technologies SA, Industria 3, 6933 Muzzano, Switzerland

**Keywords:** ophthalmic instrumentation, pupillometry, silent substitution, melanopsin, retinal assessment, retinal stimulation

## Abstract

Recent advancements in clinical research have identified the need to combine pupillometry with a selective stimulation of the eye’s photoreceptor cell types to broaden retinal and neuroretinal health assessment opportunities. Our thorough analysis of the literature revealed the technological gaps that currently restrict and hinder the effective utilization of a method acknowledged to hold great potential. The available devices do not adequately stimulate the photoreceptor types with enough contrast and do not guarantee seamless device function integration, which would enable advanced data analysis. RetinaWISE is an advanced silencing pupillometry device that addresses these deficiencies. It combines a Maxwellian optical arrangement with advanced retinal stimulation, allowing for calibrated standard measurements to generate advanced and consistent results across multiple sites. The device holds a Class 1 CE marking under EU regulation 2017/745, thus facilitating clinical research progress.

## 1. Introduction

Pupillometry combined with retinal visual stimulation is a known technique used to investigate many ophthalmic and neurological diseases, such as glaucoma [1], retinitis pigmentosa [2], age-related macular degeneration [3], ischemic optic neuropathies, and neurological disorders such as Alzheimer’s disease [4]. Chromatic pupillometry [5] is the most commonly used photopic fundus stimulation technique. It involves toggling light stimulations of different wavelengths to assess the functionality of rods, cones, and melanopsin-containing retinal ganglion cells (mRGCs), also referred to as intrinsically photosensitive retinal ganglion cells (ipRGCs) [6]. A more accurate spectral analysis of this chromatic stimuli protocol reveals significant cross-talks, thus involving the simultaneous contribution of several retina photoreceptor types to the signal triggering the pupil reaction (miosis and mydriasis). In fact, a photopic blue stimulus [7] triggers the S-cone, the mRGCs [8], and partially the M-cone [9]. Such a bulk stimuli approach is tied to an intrinsic difficulty in defining photoreceptor-selective stimuli; thus, most current studies fail to link ophthalmic diseases to specific photoreceptor dysfunctions [10].

### 1.1. Pupillometers—State of the Art

The development and validation of affordable eye-tracking devices have significantly transformed fundamental and applied studies in cognitive science and human–computer interaction [11,12], creating opportunities for both clinical and more generally commercial applications. Our literature and market analysis identified three automated pupillometers featuring reliable and objective. ColorDome, developed by Diagnosys LLC (Lowell, MA, USA) and based on Ganzfeld stimulation, combines electroretinography and pupillometry [13]. IDMED Corp. (San Antonio, TX, USA) and NeurOptics Inc. (Irvine, CA, USA) were developed to establish and validate data analysis standards through the consolidated parametric measurement of pupillary reactivity, i.e., the Quantitative Pupillometry index (QPi) and Neurological Pupil index (NPi). Such parameters have been recognized in the 2020 American Heart Association guidelines for ECC (Emergency Cardiovascular Care) and CPR (Cardiopulmonary Resuscitation), providing support for the prognosis of brain injury [14,15,16,17]. It is important to note that these previously mentioned devices have limitations in providing advanced retinal stimulations. One major limitation results from the inability to selectively stimulate different photoreceptor cell types due to the lack of control over stimulation spectral distribution. Another limitation is the fact that the retinal illuminance in Newtonian View is modulated by the pupil size.

### 1.2. Silent Substitution Technique

The retina contains three types of photoreceptor cells, including rods, cones (S, M, and L), and mRGCs, playing a crucial role in both vision and non-visual functions [18,19]. Their responses are mediated by a group of opsins, leading to distinct but partially overlapping spectral sensitivities [20]. The overlap in spectral sensitivity between the eye’s photoreceptor cells presents a challenge for selective stimulation. The use of monochromatic light cannot activate a single photoreceptor type in isolation. Inevitably, most visible-light stimulation methods will activate multiple photoreceptor cell types to varying degrees [18,20]. To overcome such difficulties, protocols employing pairs of distinct stimulation methods were introduced to characterize photoreceptors’ responses in terms of contrast (e.g., Michelson’s or Weber’s contrast). Silent substitution [21,22] is a well-known technique used to optimize the *stimulation contrast* of a selected photopigment. The silent substitution technique, a method that exploits the principle of univariance [23], allows the selective stimulation of given photoreceptor cells while silencing the others [24]. It can be used to define the spectral and intensity characteristics of two or more retinal stimuli. By switching between these stimuli, one can selectively activate the target photoreceptor cell while keeping the activation of all the others constant [21,25]. This method, by significantly advancing photoreceptor research, particularly in recent pupillometry studies, has underscored its significance and impact [26,27,28,29]. To further catalyze the adoption and use of the advanced silent substitution technique, Martin et al. developed an algorithm to assist users in calculating the optimal light configuration pairs to achieve the highest contrast by activating the targeted photoreceptor cell type selectively while silencing the other. The *PySilSub* [27] Python [30] toolbox is freely accessible, thus making silent substitution stimulation accessible to researchers and practitioners.

### 1.3. Pupillometry Combined with Silent Substitution Technique

While many laboratories have successfully developed devices capable of stimulating the eye with specific light wavelengths, enabling the use of silent substitution techniques [31,32,33], no commercial devices are currently available. Our thorough analysis of the existing literature, discussions, and debates with clinicians and researchers has highlighted a consolidated need for a new combined silenced pupillometer. This pupillometer should have a binocular arrangement, controlled retinal illuminance, and be easy to use. Such an instrument would significantly promote and improve research activities focused on the cause–effect relation between pupillometry assessments and ophthalmic or neuro-ophthalmic disorders.

This paper introduces a new binocular pupillometer that uses a Maxwellian optical arrangement [34] and an intensity- and spectrally controlled light stimulation engine to apply the silent substitution technique. This allows for the selective stimulation of different types of photoreceptor cells while simultaneously assessing eye pupil diameter and dynamics.

## 2. Materials and Methods

### 2.1. Silenced Pupillometer

**Figure 1:**
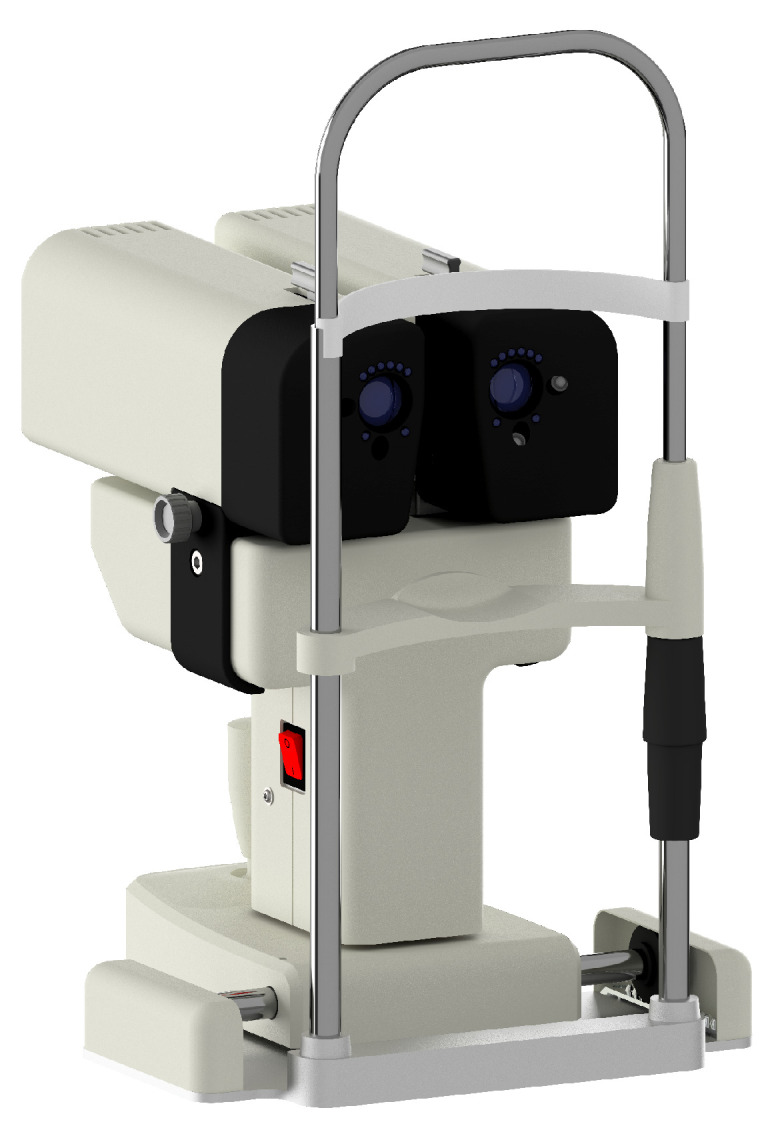
retinaWISE, a binocular silenced pupillometer equipped with three cameras allowing for simultaneous and bilateral pupil monitoring and two controlled light engines to generate silent substitution stimulation of the patient’s eye fundus. An ophthalmic joystick and a chin rest are included to align the device with the patient’s eyes.

The developed silenced pupillometer, named retinaWISE, is depicted in Figure 1. It is based on Maxwellian optical arrangement [34] and features two independent light channels to simultaneously and bilaterally stimulate the patient’s eye photoreceptor cells.

**Figure 2:**
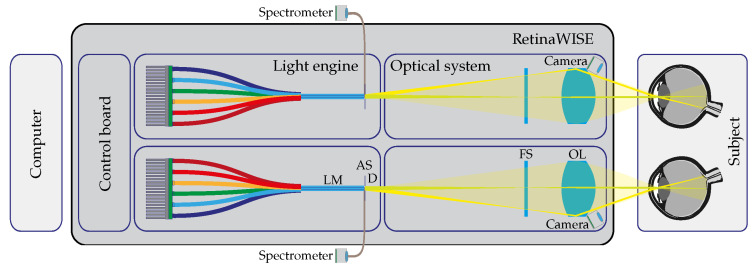
Schematic view of the retinaWISE internal components including a control board, a light mixer (LM), a light-source aperture (AS), a diffuser (D), a field stop (FS), an ophthalmic lens (OL), and cameras. The external components included a computer (PC), which was connected to the control board, and a spectrometer.

The Maxwellian optical setup depicted in Figure 2 was designed to maintain a consistent light intensity that is projected onto the retina, regardless of changes in the patient’s eye pupil diameter [34]. This was achieved by making the exit pupil of the device smaller than the smallest statistically recorded human eye pupil (see Section 3.1.3). The device was also designed to create equi-spectral retinal spots, ensuring consistent stimulation across the entire area. These features, along with spectral and intensity-controlled requirements for effective silencing, are the key characteristics of the retinaWISE system.

#### 2.1.1. Optical Setup

The optics used to stimulate the fundus of the eye were composed of two sub-systems: a light engine and an optical projection system (Figure 2). Each fundus stimulation light engine was equipped with six high-brightness LEDs (referred to in the text as LED1 to LED6 with nominal peak emission wavelengths of 420 nm, 450 nm, 470 nm, 520 nm, 590 nm, and 630 nm; SMB1 series of Roithner Lasertechnik, Austria), with the help of a light mixer (LM) and a diffuser (D). These LEDs were carefully selected (see Section 4) to generate spectral characteristics targeting the different retinal photoreceptor cell types [25]. The cameras monitoring the pupils used eight NIR LEDs (940 nm, IR7373C, Distrelec, Nänikon, Switzerland) for each eye, ensuring consistent pupillometry data without emissions in the visible range.

The optical projection system relays the internal aperture on the patient’s pupil (Maxwellian view) with a commercial ophthalmic lens (OL) (F = 32 mm).

The projection system allows for a maximum Field of View (FoV) of 60°, while the size and shape can be adjusted with a custom field stop (FS) which can be optionally added between the light source and the ophthalmic lens. Within the optical setup, a 200 μm fiber tip, polished at 45°, was placed before the internal light source’s aperture (AS) to collect part of the generated light beam. This fiber can be optionally connected to any external device, i.e., a spectrometer, allowing measurement of the light source’s spectrum and intensity without interfering with ongoing eye fundus stimulation and pupillometry measurements (see Section 2.2).

#### 2.1.2. Electronics

The Pulse Width Modulation (PWM) signals used to modulate LED light intensity were generated by utilizing the internal clock of the micro-processing unit (MCU) operating at 96 MHz. A 16-bit timer and a prescaler were used to obtain a PWM frequency of 183.1 Hz. Each LED was equipped with its dedicated current source (RCD-24-0.70, RECOM Power GmbH, Munzfeld, Austria), capable of handling a maximum output current of 700 mA which could be modulated with the aforementioned PWM signal. The PWM driver provided an 11-bit resolution for intensity control. Additionally, an optional optical filter (OD) could be integrated after the light mixer to extend the intensity range further. To maintain safety, LED currents were constrained to keep fundus illumination below the threshold for photobiological and thermal damage. This design ensured that the device’s safety was guaranteed by hardware rather than software. The control board was connected to the external PC through a USB connection, on which the retinaWISE software was installed.

#### 2.1.3. Alignment and Pupil Measurements

**Figure 3:**
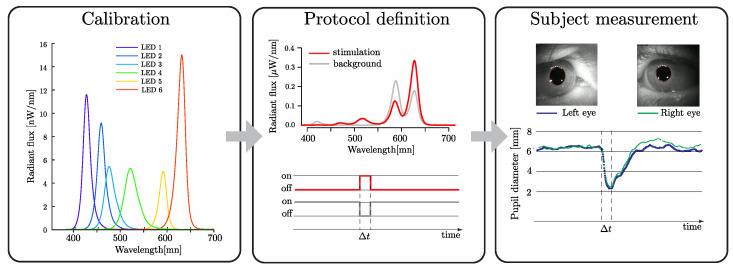
Schematic of the protocol generation and measurement process, exemplified by an mRGC silent stimulation procedure.

To assess pupil measurements, the typical procedure was as follows: Firstly, the LEDs’ emissions were calibrated using a spectrometer and powermeter. Secondly, the PySilSub toolbox [27] was utilized to create specific fundus stimulation protocols. Finally, once defined, these protocols were programmed directly into the retinaWISE microprocessor (Figure 3).

To ensure the proper alignment and monitoring of the patient’s pupil, the device integrated three cameras (Basler daA1280-54um, CHROMOS, Dielsdorf, Switzerland) all configured in a Scheimpflug optical arrangement [35]. Specifically, the right eye was monitored by two cameras: one laterally mounted and one positioned below. These images were triangulated to obtain the information allowing precise patient alignment; using the parallax technique, the exit pupil of the device was lined up with the patient’s eye pupil. The left eye was monitored by a single side-mounted camera, which could be adjusted horizontally to fit the patient’s interpupillary distance.

The RetinaWISE software, version 1.11.6, (Oculox Technologies, Muzzano, Switzerland) installed in an external PC was then executed to start the protocol, capturing and analyzing pupil data (see Figure 3). The device software assisted the operator during the alignment phase. Image acquisition was performed at the maximum frame rate and at least 30 Hz. Both side cameras were responsible for pupil measurements. OpenCV libraries were used for real-time image processing: adaptive thresholding, image inversion, blob detection, blob filtering, edge detection, filling of concave holes, and the determination of the ellipse that most closely approximates pupil edge.

### 2.2. Characterization and Calibration Measurements Setup

When using the silent substitution technique, it is crucial to calibrate the light source accurately to ensure that only one type of photopigment is stimulated while others are silenced. This can be challenging because the human eye contains multiple types of photosensitive cells, each with distinct but overlapping spectral sensitivities [9].

retinaWISE was factory-calibrated with ad hoc software to validate its performance while producing standardized reports for proper documentation. Developed in a LabVIEW environment (National Instruments Corp., Austin, TX, USA), the software automates the acquisition of customizable calibration measurements (see Section 3.1.5).

The in-depth characterization of the light engine was performed with two distinct instruments:

- **Powermeter:** The radiant flux of the system at the exit pupil was measured using a Thorlabs (Newton, NJ, USA) PM100D coupled with an S120VC optical head. The instrument is designed to sample radiant flux between 50 nW and 50 mW within the wavelength range of 200 nm to 1100 nm. It has a sensitive area of Φ=9.5 mm and a typical uncertainty of ±3% in the visible range. The sampling time was 50 ms, and readings were taken in *Amperes* to ensure precision and wavelength independence. The sensitivity of the optical head (A/W) was then used to convert the measurement to radiant intensity.
- **Fiber-Coupled Mini-Spectrometer:** The C10988MA-1 Hamamatsu MS series mini-spectrometer with its evaluation board (C11351, Hamamatsu Photonics, Shizuoka, Japan) was coupled to the integrated collecting fiber of the device, as shown in Figure 2. The sensor head is characterized by a spectral resolution of 14 nm in the range of 340 to 750 nm by using a 256-pixel CMOS linear image sensor. The spectrometer has a fixed configuration and can be controlled with a dedicated calibration software developed in LabView. For all the measurements reported in Section 3.1, the integration time was set to 20 ms, and the gain was fixed to *low*. Before each measurement, a dark reading averaged 10 readings with all LED lights turned OFF to account for external illumination.

The measurements obtained using the listed tools enabled us to characterize the system in terms of total emitted radiant flux (W), irradiance (W/cm^2^), and spectral flux (W/nm). This is in line with the methodologies presented in other research studies [36,37,38]. The system’s performance was therefore investigated in terms of stability over time, linearity, beam size at the device exit pupil, and repeatability over an extended period. The results are presented in Section 3.

### 2.3. Preliminary Experiment Setup

To further evaluate the functionality of the device, the right eyes of 6 subjects, four males and two females aged between 28 and 65, with no diagnosis of ophthalmological disorders and considered healthy persons, were stimulated using 3 different light combination protocols, namely *blue*, *red* and *melanopsin*. Each protocol was defined to activate different photosensitive cells.

The *blue* and *red* protocols were configured to use only one wavelength, i.e., only one LED, specifically LED3 (470 nm) and LED6 (630 nm). The *melanopsin* protocol was configured to target mRGCs with a Michelson contrast [39] equal to 50%, while isolating cone cells [21].

**Figure 4:**
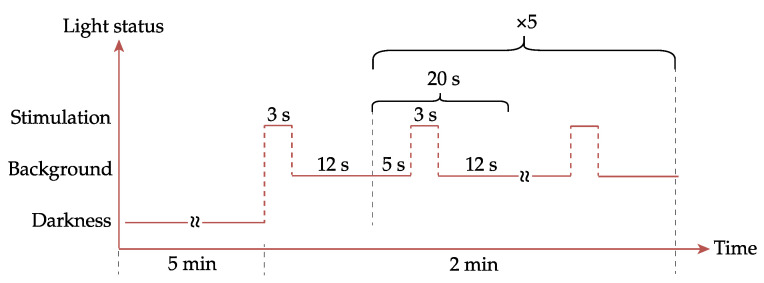
Protocol timing: for each 3 s stimulation, a 20 s window was defined, with 5 s pre-stimulation serving as a baseline and 12 s post stimulation for pupil recovery.

To obtain this, the calibration data, along with experiment parameters, were provided to the PySilSub Python Toolbox [27] to calculate two LED combinations: stimulation and background. Subsequently, the retinaWISE software constructed a time-defined protocol for the experiment, which alternated between stimulation and background, as shown in Figure 4. While calculating the LED combination, the rod’s activation was neglected due to the photopic vision luminosity range used for this protocol [7]. To address inter-observer differences in ocular physiology [40,41,42,43], the protocols involving short wavelengths, i.e., *blue* and *melanopsin*, were defined for 3 age categories (20 to 34, 35 to 49, and 50 to 70), while the settings for the red stimulation protocols were kept unchanged for all subjects.

The right eye received stimulation within a ±14° FoV with a central ±5° obscuration, while the left eye remained unstimulated. The FoV for the three protocols was selected to obtain an illuminated area on the retina equal to $A_{r}=48.7$ mm^2^, calculated based on the projection of incoming light into the pupil according to the focal length of the Walker model of the human eye [44].

For the three protocols, the background and stimulation parameters were accurately measured and are reported in Table 1.

Prior to each protocol, all subjects underwent a 5 min dark adaptation. As shown in Figure 4, each protocol lasted 120 s and contained six 20 s sequences constituted by 3 s of stimulation and 17 s of background illumination.

Finally, the three protocols were loaded into the control board of the retinaWISE to conduct preliminary experiments with the subjects.

## 3. Results

### 3.1. Characterization and Calibration Results

To assess the device’s performance, several characterization measurements were carried out focusing on the critical aspects of these instruments [22], evaluating the reliability and the quality of the generated light stimuli.

#### 3.1.1. LEDs’ Emission Stability as Function of Time

**Figure 5:**
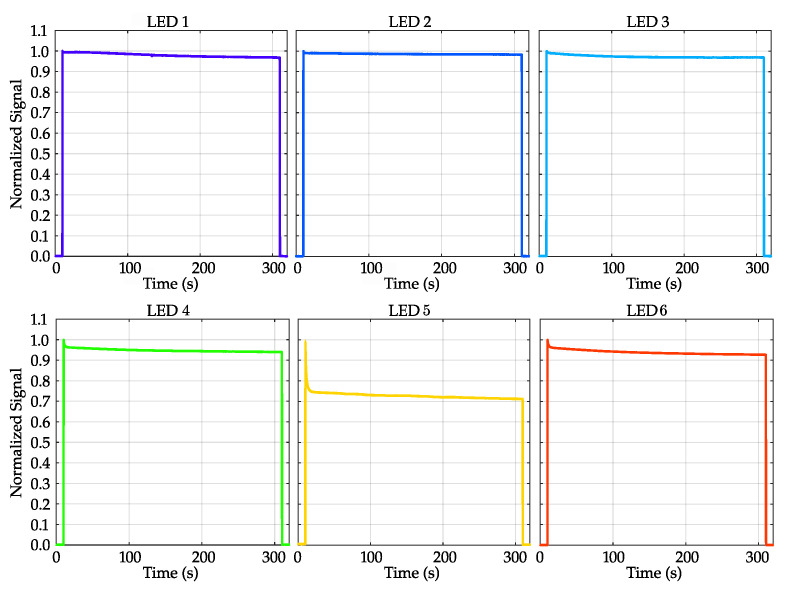
Stability test performed on each of the six LEDs with maximum output current (LR = 1). Each curve was normalized to the maximal peak value of the respective LED. The curves are color-coded to match the LEDs.

The stability of the total radiant flux for each LED was assessed by activating each of the six LEDs with a light ratio (LR) of 1 (100% duty cycle (DC)) for 5 min. Throughout this period, as shown in Figure 5, the fluctuation regarding the total radiant intensity was measured with Thorlabs PM100D at the device’s exit pupil and the maximal peak value of the respective LED was normalized.

When evaluating LED performance at full power for five minutes (Figure 5), blue-light LEDs retained over 95% of their initial emitted radiant power, while red and green LEDs remained in the 93–95% range. However, the radiant flux of the yellow LED declined rapidly, stabilizing at 72%, probably attributable to the lower quantum efficiency in this wavelength range [45].

#### 3.1.2. LEDs’ Linearity

**Figure 6:**
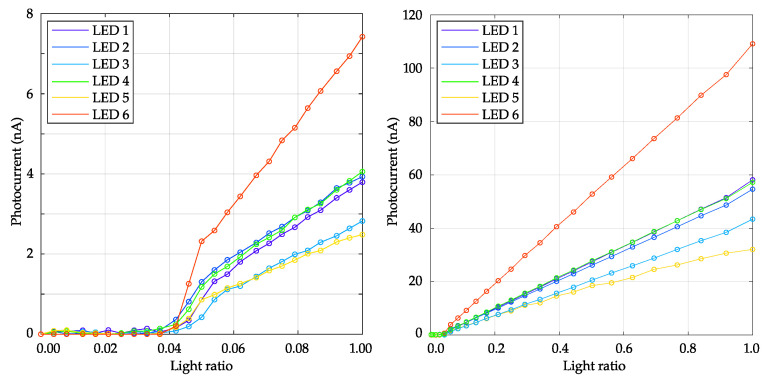
On the left, for each LED, the radiant flux, in photocurrent (nA), was measured for a random increase in the light ratio (LR) from 0 to 0.1 with 25 steps of resolution. On the right, measurements were performed by setting the LR randomly between 0 and 1 with 25 steps for each LED. The curves are color-coded to match the LEDs.

Characterizing the linearity of the emitted light intensity by quantifying the radiant flux, measured by the powermeter in nA, as a function of the light ratio (LR) or duty cycle (DC), is crucial to the generation of spectral distribution for the silent stimulations of photopigments [21]. To assess overall linearity, two measurements were performed, measuring the radiant flux for each LED by randomly setting the LR between 0 and 10% and between 0 and 100%. As depicted in Figure 6 the radiant flux relationship appears predominantly linear, characterized by a small offset common to all LEDs, where the turn-ON starts at around 4% DC. This offset, probably due to LEDs driven with very short pulses, can be taken into account for the generation of the protocols through silent substitution optimization with the *PySilSub* Python suite [27].

#### 3.1.3. Stimulation Beam at Device’s Exit Pupil

**Figure 7:**
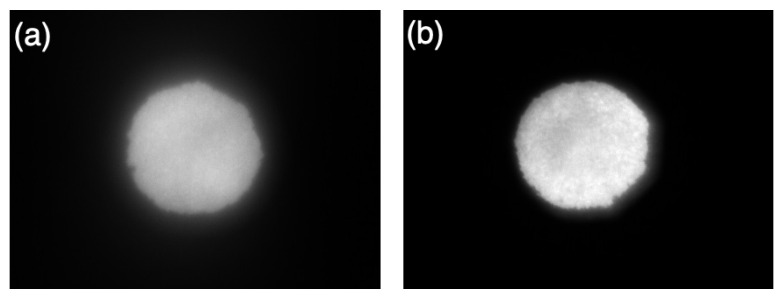
Representative exit pupil image at different FoVs: (**a**) 60°and (**b**) 10°.

To characterize the quality of the light entering the patient’s eye, measurements of the shape and intensity distribution of the stimulating beam at the device’s exit pupil were performed. Using a standard commercially available imaging system (Sony ICX204AL CMOS sensor (Sony Corporation, Tokyo, Japan)), the beam diameter and sharpness were assessed at two different FoVs: a maximum FoV of 60° and a 10° FoV, achieved with a small circular FS. As expected, at the 60° FoV, the edge of the exit pupil image was blurred, resulting in a slightly larger beam diameter compared to the 10° FoV (Figure 7).

At the 60° FoV, the exit pupil area was 0.406 mm^2^ (width: 0.720 mm, height: 0.730 mm), while at the limited 10° FoV, the area was slightly smaller with an area of 0.380 mm^2^ (width: 0.716 mm, height: 0.678 mm) as reported in Table 2.

#### 3.1.4. Light Emission Repeatability over Time

To assess the device’s consistent performance and reliable measurements, light emission repeatability was evaluated. Executing a stimulation protocol with two light combinations, namely background and stimulation, required that both light intensity and spectral distribution were consistently repeatable. While a slight variation in retinal illumination may be generally acceptable, maintaining a constant ratio between the two spectral intensities is critical over extended periods.

This ratio ($R_{SB}$) was defined as follows:

(1) $$R_{SB}=\frac{\sum_{i}LR_{i}\left(\mathrm{stimulation}\right)\cdot \Phi_{i}}{\sum_{i}LR_{i}\left(\mathrm{background}\right)\cdot \Phi_{i}}$$

where *i* corresponds to the *i*-th LED and LR to the light ratio within [0,1]. LR represents the numerical values corresponding to the DC of PWM signals that fed the driving electronics, while $\Phi_{i}$ were values that depended on physical conditions such as the temperature of the electronics, aging of the LED, degradation of optical components, and other environmental variables.

The ratio $R_{SB}$ depends on the selected protocol, specifically on the intensity setting of each LED. To evaluate the long-term (60-day) stability of the $R_{SB}$ ratio, six $R_{SB}$ ratios corresponding to six protocols were defined. Five protocols included one LED (LEDs 1 to 5) as the stimulation source and the LED6 as the background, with the light ratio set to LR=0.4 for all LEDs. The sixth protocol was a combination of two *metamers* (B and S). Table 3 gives the LRs used for the two combinations.

**Figure 8:**
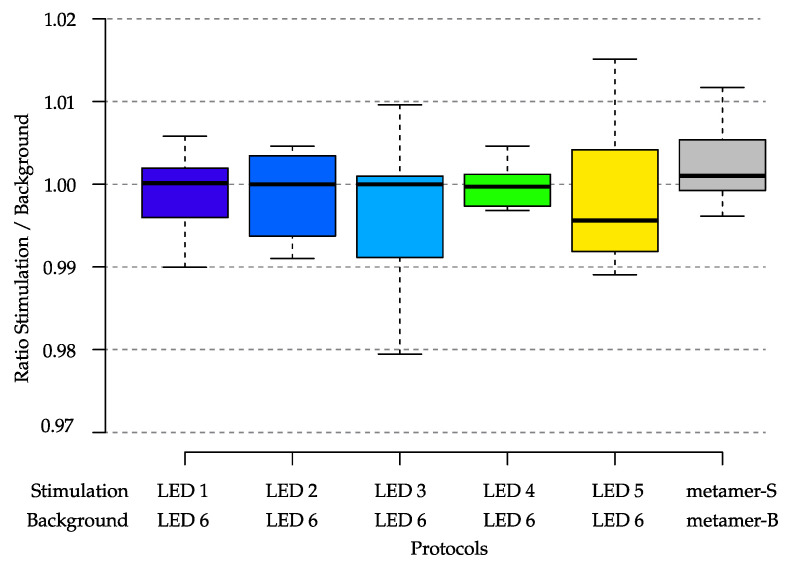
Variations in the light emission ratios between stimulation and background. Measurements were performed over 60 days without controlling the temperature of either the device or the measurement environment. All boxes except the last one were color-coded to match the stimulation LEDs.

Each experiment was conducted under worst-case conditions, without warming up the device and in an environment with temperatures ranging from 18 °C and 22 °C over the 60 days. Figure 8 shows the obtained $R_{SB}$ ratios of the selected LED combinations. The mean ratios of LED1 to LED4 exhibited very stable values, while LED5 revealed a ratio of 0.996. This variation in the ratio can be related to the lower stability of the yellow LED shown in the previous section. Furthermore, the highest variance was observed for LED3, with values ranging from 0.98 to 1.01. The metamer combinations displayed a ratio close to 1.002.

#### 3.1.5. Spectral Flux Measurements

**Figure 9:**
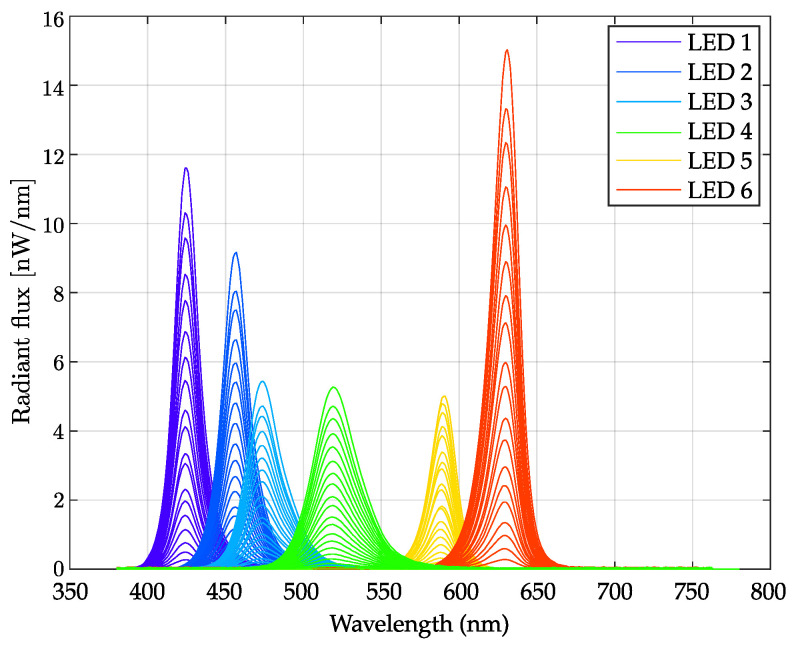
Calibration Report: spectral flux measurements obtained by combining spectral distribution collected with a C10988MA Mini-spectrometer (Hamamatsu Photonics, Shizuoka, Japan), and total radiant flux measured with the optical powermeter PM100D, (Thorlabs inc., Newton, NJ, USA). In the graph, the curves were collected by changing the intensity of each LED with 25 resolution steps in a random fashion.

The calibration process is crucial for obtaining reliable measurements, since the internal components of the specific retinaWISE device unit may have a poor manufacturing tolerance. Spectral flux was collected by activating each LED individually at specific power levels. This process was repeated for each combination of LEDs and 25 input power values, resulting in a total of 150 measurements. The combined data are shown in Figure 9.

Following the procedure described in Section 2.3, the factory calibration report was used as an input in the PySilSub Toolbox [27] to generate silent substitution protocols that were tuned to the specific device’s light spectral stimulation. Inside the application suite, both the observer pre-reception filters and device FoV can be optionally adjusted for a particular experiment.

### 3.2. Preliminary Experimental Results

**Figure 10:**
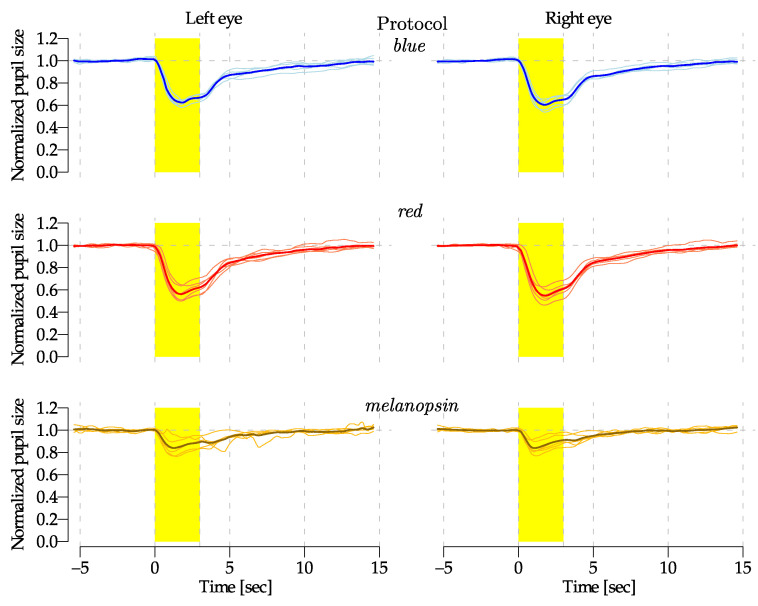
Normalized pupil size (diameter) variation in response to *blue* (470 nm), *red* (630 nm), and *melanopsin* stimulation, from top to bottom. The stimulation was applied to the right eye while both eyes were measured simultaneously. The thick line represents the mean of individual curves (thin lines). The yellow box indicates the three-second stimulation period.

Each protocol was repeated three times for each subject. The pupil measurements were analyzed by superimposing the final five out of six stimulations. The results obtained for the *blue*, *red*, and *melanopsin* protocols are reported in Figure 10. Single responses were averaged after having normalized the pupil size (diameter) to its corresponding baseline. The bold lines express the mean value of the recorded pupil size, while the light lines display the averaged measurements of a single subject. Figure 10 illustrates the pupil size response for both eyes and each applied protocol. The *red* and *blue* protocols showed clear variations in pupil diameter, while the *melanopsin* protocol exhibited a slighter pupil size variation.

For each subject and protocol type, the escape velocity was estimated through a linear fit of the pupil diameter between the maximum constriction and the end of the stimulation (Table 4).

A general linear hypothesis and multiple comparisons for parametric models using R-statistics [46] found that the difference between the *blue* and *red* escape velocities was significant (p=0.0286), as well as the difference between *red* and *melanopsin* stimulation (p<0.001), but not that between *blue* and *melanopsin* (p=0.1891).

## 4. Discussion

This study aimed to characterize retinaWISE, a medical device designed for advanced retinal stimulation and precise pupillometry to assess retinal function more effectively.

**Figure 11:**
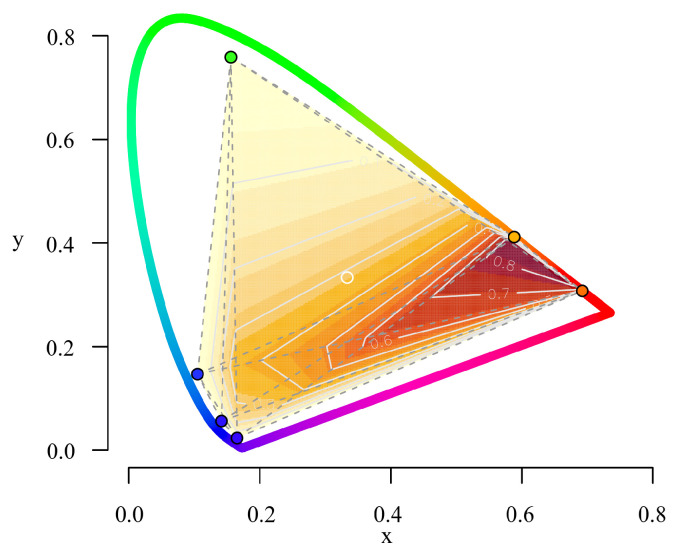
Level maps of the Michelson contrast for melanopsin-containing retinal ganglion cell silent stimulation over the CIE chromaticity diagram for the selected six primary peak wavelengths simulated with a Gaussian spectrum profile.

Starting from an in-depth review of existing pupil stimulation devices [5,31], we developed retinaWISE to cover the stimulation of most of the human visual spectrum while maintaining simplicity and reliability by incorporating six primary wavelengths. Given the close spectral overlap between S-cones and melanopsin-containing retinal ganglion cells (mRGCs) [9,43], three specific wavelengths within the blue spectrum were chosen to optimize the silencing of such cells. Moreover, despite the challenges in sourcing LEDs with emissions between green and red, the selection of a yellow LED was crucial for effectively stimulating mRGCs [25]. This configuration enabled a theoretical Michelson contrast for mRGCs of up to 87% (see Figure 11), thus more than most other previously developed devices for research purposes [31].

Using only four primary light sources to stimulate mRGCs is possible; this typically leads to a pattern where two LEDs operate at very low intensity and the other two at higher intensity for one combination, while this pattern alternates for the other combination [47,48]. Incorporating six LEDs, however, significantly enhances the device’s flexibility, allowing for the finer tuning of light spectra pairs. Another key aspect of retinaWISE concerns the possibility of addressing the non-uniform distribution of photoreceptors along the retina [49,50]. This has been made possible thanks to the Maxwellian-like optical design combined with the possibility of adding custom field stops (see Figure 2(FS)), which enables retinaWISE to selectively stimulate specific retinal regions while maintaining the other in darkness. However, achieving effective stimulation requires maintaining stable eye positioning and consistent gaze direction. Future applications of this device will include detailed studies designed to evaluate the differential contributions of various retinal zones through the utilization of differently shaped field stops. This will involve calibrating our device’s stimulation parameters to account for regional variations in photoreceptor and pigment epithelium density.

The characterization of retinaWISE revealed important insights into its performance. The stability of LED emission over a prolonged period (5 min) demonstrated robust performance (see Figure 5), with the blue LEDs maintaining over 95% of their initial radiant power, while the red and green LEDs remained within the 93–95% range. However, the yellow LED exhibited a significant decline to 72%, likely attributable to lower quantum efficiency in this wavelength range [45]. So far, when utilizing the yellow LED to silence one photoreceptor, it is important to consider its high intensity and extended ON time. To address such limitations, the PySilSub Toolbox provides the possibility of calculating LED light ratio combinations while limiting the intensity of one or more sources arbitrarily, albeit at the cost of reduced maximum contrast. While one possible solution is to reduce the peak current on these LEDs, for future improvements we will focus on temperature-compensated LED drivers or optical feedback driver-based systems [51].

Assessments of LED linearity (see Figure 6) revealed predominantly linear relationships, albeit with minor offsets at low-duty cycles or light ratio (LR) values. While a perfectly linear relationship between LR and light intensity would benefit easier and faster calculations for silent substitution protocols [25], small non-linearities are not considered critical as they can be easily compensated through the calibration process of the device.

The optical setup implemented in our device (see Section 2.1.1) proved to be capable of keeping a consistent shape of the output light stimuli. The measurements reported in Section 3.1.3 indicated that while the exit pupil size and shape varied within different FoVs, the aspect ratio and roundness were consistent (Table 2). Since the maximal measured device’s exit pupil diameter was smaller than 0.8 mm, and considering that the smallest observed human pupil has a diameter of about 2 mm [52], retinaWISE is compliant with the Maxwellian optical arrangement.

Concerning the long-term repeatability (see Section 3.1.4), as observed over 60 days, the stimulation-to-background ratio ($R_{SB}$) remained stable for most of the LEDs, with minor variations observed (see Figure 8). The yellow LED displayed a slightly lower stability, aligning with its earlier noted rapid decline in radiant flux (see Figure 5). This stability is crucial for long-term studies where consistency in light stimulus is necessary to ensure reliable and repeatable results. Adequate warm-up times and controlled environmental conditions could further enhance the stability of the light stimuli [22].

The calibration of the device (see Section 3.1.5) proved to be a crucial step for the configuration of silent substitution protocols. The ad hoc developed software (see Section 2.2) proved to be a valuable tool for automating both factory calibrations and daily calibrations which can be easily set up by the final user. Despite the mini-spectrometer used for the calibration having a limited resolution of 14 nm, comparisons with higher-resolution devices (CCD linear sensor C5966-31 and Hamamatsu PMA-11, <3 nm FWHM) demonstrated minimal discrepancies in our laboratory tests. While a more sophisticated device could be used in the future through plug-and-play connection with internal optical fiber (see Figure 2), the mini-spectrometer’s resolution was accurate enough to ensure reliable and repeatable results.

The preliminary results from six subjects showed distinct variations in pupil diameter in response to blue and red light stimuli, while the *melanopsin* protocol induced minimal changes. These findings are consistent with the known spectral sensitivities of different photoreceptors [9]. The tests conducted primarily aimed to validate the accuracy of the pupil measurements, thus focusing on two simple protocols and one silent substitution targeting mRGCs. Given that mRGC stimulation with only blue light is often unpleasant due to high retinal illuminance, a low illuminance was employed for both cases (see Table 1). As expected, the low light exposure (≈13 log photons per cm^2^ per second) resulted in similar post-illumination pupil responses for both *blue* and *red* stimulation [5]. Despite the short three-second stimulation period, the escape velocity confirmed the slower response of S-cones compared to L-cones (see Table 4), confirming previous studies [53,54].

In contrast, the similarity observed between the *blue* and *melanopsin* protocols is probably related to the *blue* protocol, which utilized a single 473 nm LED. This wavelength stimulates both S-cones and mRCGs to a similar degree, being the S-cone-opic Equivalent Daylight Illuminance (EDI) 20% less than melanopic EDI during the stimulation at this wavlength [18,55]. Moreover, our *melanopsin* protocol achieved a 22% contrast in stimulating S-cone, whereas it was 51% for mRGCs [56,57,58].

These preliminary results along with the extended characterization tests confirm the device’s capability to generate specific and reproducible light stimuli that align with known photoreceptor responses. Future studies should expand on these findings by incorporating different protocols and a larger subject cohort to further validate the device’s performance. The presented pupillometer has the potential to be a valuable tool for the diagnosis of eye diseases and in the field of neuro-ophthalmology [59,60], the study of color vision [61], and the circadian cycle [62]. Additionally, it can contribute to investigations into the role of photoreceptors and mRGCs in other visual functions, such as visual acuity [63] and contrast sensitivity [64].

## 5. Conclusions

Since the market offers few standard instruments, numerous scientific publications studying retinal function exploit in-house-developed devices [31,32]. Our solution, which combines a Maxwellian optical arrangement with a novel binocular light-based stimulation system, enables independent targeting of single-type photopigments. This capability not only advances retinal research through the use of the silent substitution technique but also serves as a model for chromatic pupillometry. This Class 1 certified device is already available on the market and many different clinical validation tests are in the pipeline. This device facilitates large-scale pupillometry testing, enabling researchers to compare results across different groups for assessing the inner and outer retina using a standardized instrument [65].

## Figures and Tables

**Table 1:** Protocol light characteristics: chromaticity (*x*, *y*), radian flux (μW) at entrance pupil, and retinal illuminance (lm/m^2^ and log photons/cm^2^/s).

	*Blue*		*Red*		*Melanopsin*	
Parameters	Background	Stimulation	Background	Stimulation	Background	Stimulation
Chromaticity (*x*, *y*)	(0.131, 0.208)	(0.105, 0.197)	(0.658, 0.316)	(0.659, 0.315)	(0.585, 0.379)	(0.589, 0.387)
Pupil Radiant Flux (μW)	0.023	0.935	0.017	2.610	4.534	5.282
Retinal Illuminance (lm/m^2^)	0.184	2.034	0.235	53.084	34.772	33.424
(log photons/cm^2^/s)	11.06	12.67	11.25	13.22	13.45	13.52

**Table 2:** Exit pupil size measurement at different FoVs.

FoV	Area (mm^2^)	Width (mm)	Height (mm)	Aspect Ratio	Roundness
FULL (60°)	0.406	0.720	0.730	1.033	0.968
LIMITED (10°)	0.380	0.716	0.678	1.057	0.946

**Table 3:** Light ratios of the two combinations used while stimulating the mRGCs.

	LED1	LED2	LED3	LED4	LED5	LED6
metamer-B	0.721	0.100	0.100	0.100	0.699	0.100
metamer-S	0.100	0.100	0.899	0.105	0.100	0.899

**Table 4:** Mean of pupil escape velocity and recovery time for different light stimulations for all subjects and both eyes. The recovery time was obtained by fitting the normalized pupil data.

	*Blue*	*Red*	*Melanopsin*
Escape velocity (mm/s)	0.235±0.093	0.346±0.086	0.155±0.129
Time to recover (s)	11.4±6.0	8.5±2.2	3.5±2.0

## Data Availability

Data and anonymous preliminary measurements are accessible by submitting a formal and scientifically sound request to the corresponding author.

## Abbreviations

The following abbreviations are used in this manuscript:

mRGCs	Melanopsin-Containing Retinal Ganglion Cells
QPi	Quantitative Pupillometry index
NPi	Neurological Pupil index
NIR	Near InfraRed
ECC	Emergency Cardiovascular Care
CPR	Cardiopulmonary Resuscitation
ipRGCs	Intrinsically Photosensitive Retinal Ganglion Cells
PLR	Pupillary Light Reflex
PWM	Pulse Width Modulation
FoV	Field of View
LR	Light Ratio
EDI	Equivalent Daylight Illuminance
